# OP42 A Budget Impact Analysis Of The Introduction Of Noninvasive Prenatal Testing To Korean National Health Insurance

**DOI:** 10.1017/S0266462324001041

**Published:** 2025-01-07

**Authors:** Hyerin Seo, Jeonghoon Ahn, Doyeon Jin, Hyorim Lee

## Abstract

**Introduction:**

This study assessed the budget impact (BI) of introducing noninvasive perinatal testing (NIPT) to the Korean National Health Insurance (KNHI). It aims to provide useful economic evidence on reimbursement decisions on prenatal screening. Since NIPT is widely used in Korea as a noncovered service, BI can be reliably estimated from real-world data (RWD).

**Methods:**

In this BI analysis, we derived the total number of pregnant women from 2017 birth statistics (n=358,000) and its distribution of singleton births (96.1%) and multiples (3.9%). The age distribution of pregnant women was 29.4 percent for 35 and above, and 70.6 percent for below 35, according to the 2017 statistics. For NIPT costs, we considered a price range of KRW357,000 (USD260) to KRW715,000 (USD522) from a 2018 survey. To evaluate the BI of introducing NIPT, we examined eight scenarios based on NIPT cost sharing of 30 percent and 70 percent, NIPT price, and whether to include pregnant women aged less than 35 years old.

**Results:**

BI for KNHI was estimated as KRW6 trillion (USD4 billion) to approximately KRW56 trillion (USD40 billion). Scenario seven, targeting older pregnant women with serum screening high risk with NIPT price at the lower end and payer coverage of 30 percent, shows the lowest annual BI of KRW6,115,813,866 (USD4,469,516); scenario two, covering all older pregnant women and younger high-risk cases with NIPT price at the higher end and payer coverage of 70 percent, exhibits the highest burden at KRW56,240,636,611 (USD41,091,993).

**Conclusions:**

Our BI analysis of introducing NIPT to KNHI can serve as essential data for estimating insurer burdens during the potential transition of NIPT to provisional or selective coverage. In the absence of prenatal diagnostics cost-effectiveness research in Korea, our findings provide crucial evidence for establishing relevant reimbursement criteria, addressing a gap in current research and supporting evidence-based policymaking.

